# Supplementary figures and images for: Applying the Techniques of Materials Science towards an Understanding of the Process of Canine Intervertebral Disc Degeneration
Source: Animals (Basel). 2024 Sep 13;14(18):2665. doi: 10.3390/ani14182665 (PMC11428788; doi:10.3390/ani14182665)

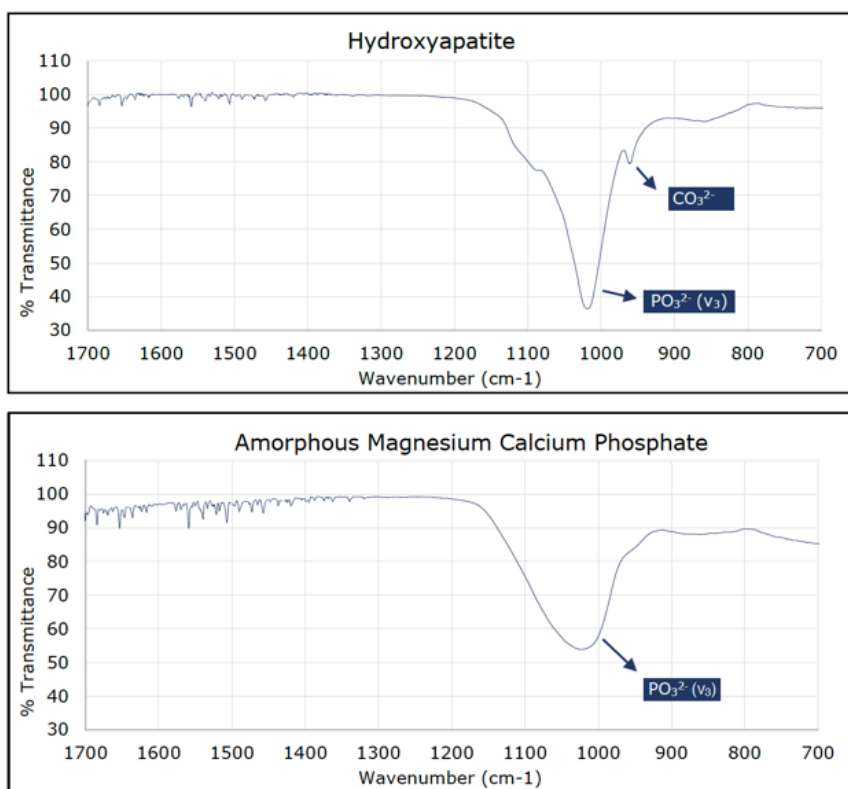

Supplementary Figure 1: FTIR smoothed spectra of test materials.

Supplement: Supplementary file 1 [file animals-14-02665-s001.zip › animals-3160716-supplementary.pdf]
